# An Epitome of Inorganic Chemistry and Physics

**Published:** 1904-01

**Authors:** 


### Bibliography.

An Epitome of Inorganic Chemistry and Physics. By A. McGlannan, M.D., of the College of Physicians and Surgeons, Baltimore. In one 12mo volume of two hundred and sixteen pages, illustrated with twenty engravings. Lea Brothers & Co., Publishers, Philadelphia and New York.

This is another of the Epitome series, and the author says of it, "The purpose is to set forth the accepted and proved facts, forming the basis of the sciences in a manner which, in the author's opinion, will serve best for the clear and easy understanding by the students."

The subjects embraced in this epitome cover physics and inorganic chemistry, hence heat, light, electricity, magnetism, together with those belonging to the latter subject, are very clearly set forth with commendable brevity, yet with a degree of thoroughness that compares favorably with larger works upon these subjects. The author claims no originality, his idea being to bring the essentials in a form adapted for reference and an aid to study.

The diagram of "Metric and Apothecary System Equivalent," giving "comparative scales, showing at a glance the exact equivalent of ordinary weights and measures in those of the metric system and *vice versa*," will prove to the student a ready means of immediate comparison, much more satisfactory than the ordinary table or methods of computation usually given.

